# Ectopic relapse of IgG4-related disease presenting as IgG4-related sclerosing cholecystitis: A case report and review of literature

**DOI:** 10.1097/MD.0000000000013868

**Published:** 2018-12-28

**Authors:** Keisuke Ishigami, Masahiro Shitani, Yasutoshi Kimura, Tadashi Hasegawa, Yoshiharu Masaki, Ayako Ito, Noriyuki Akutsu, Motohisa Yamamoto, Masayo Motoya, Shigeru Sasaki, Hiroki Takahashi, Ichiro Takemasa, Hiroshi Nakase

**Affiliations:** a Department of Gastroenterology and Hepatology; b Department of Surgery, Surgical Oncology and Science; c Department of Pathology; d Department of Rheumatology, Sapporo Medical University School of Medicine, Sapporo, Japan.

**Keywords:** autoimmune pancreatitis, IgG4-related cholecystitis, relapse

## Abstract

**Rationale:**

Immunoglobulin (Ig) G4-related disease (IgG4-RD) is a chronic inflammatory disorder characterized by high levels of serum IgG4, swollen organs with fibrosis and abundant infiltration of IgG4-positive plasmacytes.

**Patient Concerns:**

An 82-year-old male visited our hospital for an evaluation of a pancreatic enlargement and a bilateral submandibular adenopathy. Further investigation revealed elevation of serum IgG4 and bilateral lacrimal submandibular adenopathy. We diagnosed him with IgG4-related disease (IgG4-RD) and started administration of corticosteroid (CS) therapy. Both pancreatic enlargement and adenopathy rapidly improved; however, there was a new occurrence of diffuse wall thickening of the gallbladder during CS treatment.

**Diagnosis:**

Radiological examination revealed diffuse wall thickening of the gallbladder, and its inner layer was smooth and homogenous. These findings suggested an inflammatory change, but the possibility of malignancy could not be excluded.

**Interventions:**

The patient underwent laparoscopic cholecystectomy for a pathological diagnosis.

**Outcomes:**

Histological examination revealed a transmural infiltration of IgG4 positive plasma cells and dense fibrosis. The patient was pathologically diagnosed with IgG4 related cholecystitis presenting as an ectopic relapse.

**Lessons:**

There are 2 major types of IgG4-related cholecystitis, a diffuse wall thickening type and a mass formation type. It is sometimes difficult to differentiate IgG4-related cholecystitis with gallbladder cancer.

Corticosteroid (CS) is effective for induction of remission; however, we sometimes encounter disease relapse after reduction of CS dose. We should be mindful that some patients may relapse with new organ involvements even if the primary site and serum IgG4 level are well controlled.

## 1 Introduction

Immunoglobulin (Ig) G4-related disease (IgG4-RD) is becoming increasingly recognized as an emerging disorder characterized by elevated serum IgG4 levels, multiple organ involvement with storiform fibrosis and abundant infiltration of IgG4-positive plasmacytes.^[1–3]^

Autoimmune pancreatitis (AIP) is part of a disease called IgG4-RD that often affects multiple organs including bile ducts, salivary glands, kidney and lymph nodes. IgG4-related sclerosing cholecystitis involves the gallbladder,^[4]^ and has been reported to present as either diffuse thickening of the gallbladder wall or a localized mass mimicking gallbladder cancer. The standard treatment of IgG4-RD is oral administration of corticosteroid (CS), but surgical resection is sometimes performed because of the difficulty in differentiating IgG4-RD from malignancy. About 20% to 30% of IgG4-RD present with a relapse during the reduction of CS, however, how and where disease relapse occurs has not yet been established.

We report a case of ectopic relapse of IgG4-RD presenting as IgG4-related cholecystitis during oral CS treatment.

### 1.1 Patient consent statement

A written informed consent for the publication of patient data was obtained from the patient.

## 2 Case report

**Figure 1:**
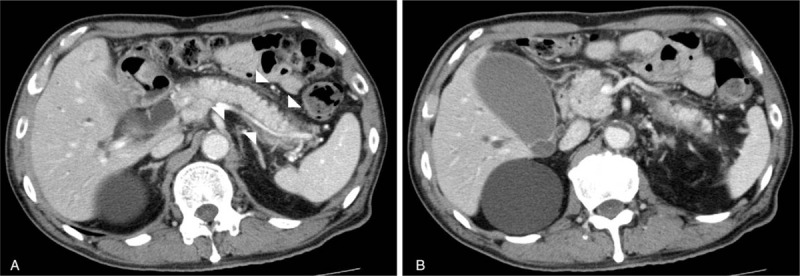
An 82-year-old man with autoimmune pancreatitis. (A) Contrast-enhanced CT showed diffuse pancreatic swelling with capsule-like rim (white arrowhead). (B) Gallbladder enlargement and intrahepatic bile duct dilation caused by constriction of the common bile duct.

An 82 year-old male visited our hospital for an evaluation of a pancreatic enlargement and a bilateral submandibular adenopathy. He had no abdominal symptom complaints. CT image showed diffuse swelling of the pancreas with a capsule-like rim (Fig. 1A) and intrahepatic bile duct dilatation and gallbladder enlargement (Fig. 1B). Serum IgG4 level had elevated to 943 mg/dL; (normal range, 4.8–105.0 mg/dL). Tumor markers, carcinoembryonic antigen (CEA), and carbohydrate antigen 19-9 (CA19-9) were within normal range.

The patient was diagnosed as IgG4-RD because of high serum IgG4 levels and organ involvement (bilateral lacrimal submandibular adenopathy and diffuse enlargement of pancreas). The patient was started on 30 mg/day of CS therapy.

Bilateral lacrimal submandibular adenopathy and pancreatic enlargement improved after initial CS treatment. Serum IgG4 level decreased to 315 mg/dL at 4 weeks after CS treatment.

During the tapering of CS, at a dose of 8 mg/day, abdominal CT revealed diffuse wall thickening of gallbladder, although this patient did not show any abdominal symptom. Serum IgG4 level was 299 mg/dL. Laboratory data showed that white blood cell counts and C-reactive protein levels were normal.

**Figure 2:**
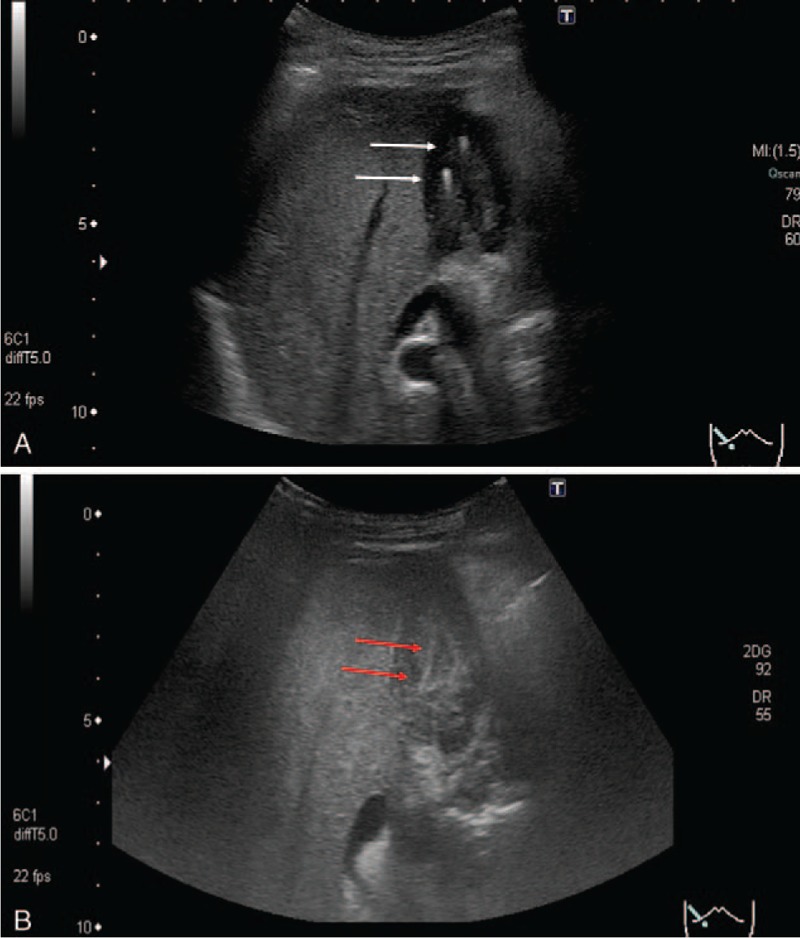
(A) Sonogram showed symmetrical wall thickening with echogenic foci (white arrow). (B) After intravenous Perflubutane injection, the inner layer of the gallbladder was enhanced (red arrow).

**Figure 3:**
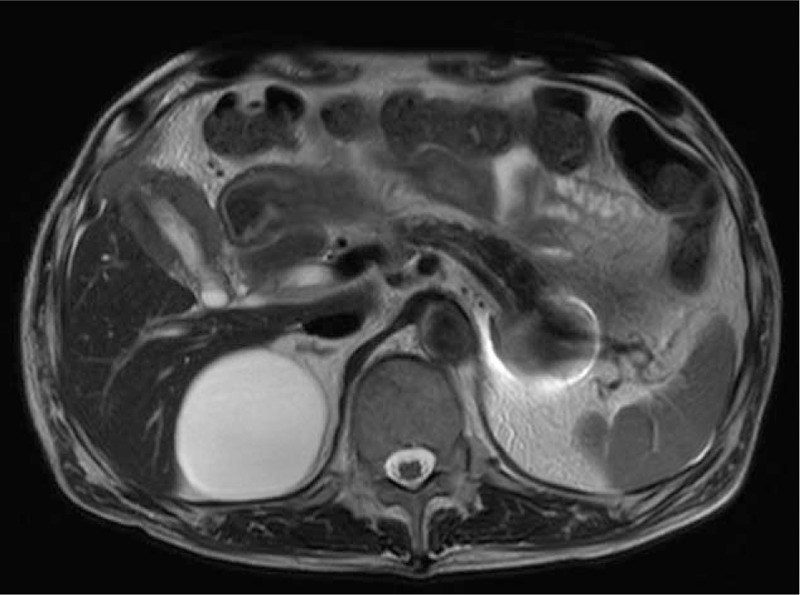
T2-weighted MR imaging revealed homogeneous thickening wall structure with hyposignal intensity. Gallbadder stones were not observed.

Abdominal ultrasonography showed symmetric wall thickening with echogenic foci (Fig. 2A, white arrow). The inner layer of the gallbladder wall was enhanced by intravenous perflubutane (Sonazoid, a second generation ultrasonographic contrast agent) injection. The surface of inner layer was smooth and laminated (Fig. 2B, red arrow). T2-weighted MR image showed gallbladder wall thickening and its inner signal was homogeneous, and high signal intensity spot indicating Rokitansky–Aschoff sinus was not detected (Fig. 3). Inflammatory changes such as xanthogranulomatous cholecystitis were strongly suspected; however, the possibility of malignancy could not be excluded, and we therefore performed cholecystectomy.

**Figure 4:**
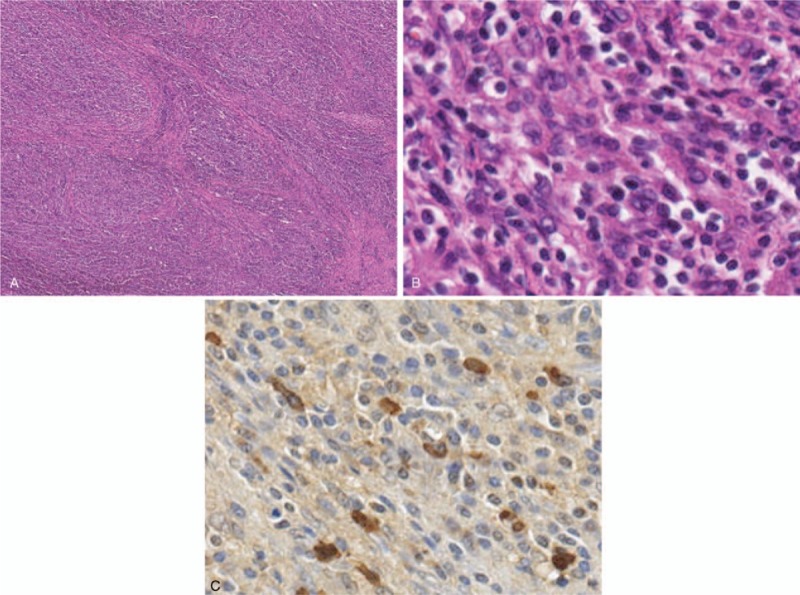
**(A)** Microscopically, the thickened gallbladder wall showed diffuse fibrosis. (H-E staining, ×25) (B) (C) Transmural lymphoplasmacytic infiltration and abundant IgG4 positive plasma cells were observed (B: H-E staining, ×200, C: immunostaining for IgG4, ×200). No evidence of malignancy was observed.

Histological examination of surgical specimens showed a transmural infiltration of plasma cells and diffuse fibrosis without malignant cells. Immunohistochemical staining revealed abundant IgG4 positive plasma cells (>30 per high-power field, IgG4/IgG ratio > 90%) (Fig. 4).

The patient was finally diagnosed with IgG4-related sclerosing cholecystitis. After operation, the patient has continued 6 mg of PSL for 3 years, and is free of relapse.

## 3 Discussion

We herein reported a case of diffuse wall thickening of the gallbladder that abruptly appeared in an IgG4-RD patient who had neither any relapse of the primary pancreatic lesion nor elevation of serum IgG4 level. We finally diagnosed the gallbladder lesion as ectopic relapse of IgG4-RD from surgical specimens. IgG4-related cholecystitis shows diffuse wall thickening and focal mass formation mimicking gallbladder cancer.

CS is effective for induction of remission, however, about 20% to 60% of cases relapse after reduction of CS dose.^[5–7]^ Yamamoto et al^[8]^ reported that 50% of IgG4-RD patients presented with new organ involvements at relapse. However, the specific organ in which IgG4-RD tends to recur remains unclear. Generally, serum IgG4 concentration at the time of diagnosis was reported to correlate with the increase in number of organs affected by the disease, more extensive organ involvement and shorter time to disease relapse.^[9–11]^ On the other hand, there have been several reports that IgG4 related lesions appeared in different sites from the primary site despite no elevation of serum IgG4 level, which is similar to our current case. Thus, we could not predict recurrence of IgG4-RD based on change in serum IgG4 level alone. For example, circulating plasmablasts and non-aspartic acid residue at position 57 of HLA-DQβ1 were reported as new biomarkers to predict disease relapse, however, the usefulness of these biomarkers has not been established yet.^[12–15]^

In this regard, identification of a new biomarker of predicting metachronous and ectopic recurrence of IgG4-RD is needed.

IgG4-related sclerosing cholecystitis involves the gallbladder.^[4]^ A systemic literature search was conducted to identify cases regarding IgG4-related sclerosing cholecystitis through PubMed search (1980 to February 2018), with following keywords: “IgG4” OR “Immunoglobulin G4” OR “autoimmune”, “cholecystitis” OR “gallbladder,” 197 articles were initially identified. After screening and reviews of the reference lists, 12 cases of IgG4-related sclerosing cholecystitis have been previously reported (Table 1).^[16–25]^ Including our case, 5 of 13 cases showed diffuse wall thickening, while the remaining eight cases presented with focal mass formation mimicking gallbladder cancer. Previous reports have indicated the difficulty in diagnosing IgG4-related cholecystitis for 2 major reasons; only 6 of 13 cases showed serum IgG4 levels of ≧135 mg/dL at the point of diagnosis, for pathological diagnosis, the usefulness of endoscopic ultrasonography fine-needle biopsy (EUS-FNA) is limited to the mass formation type. From this perspective, a distinctive morphological finding of IgG4 related cholecystitis is needed.

**Table 1:**
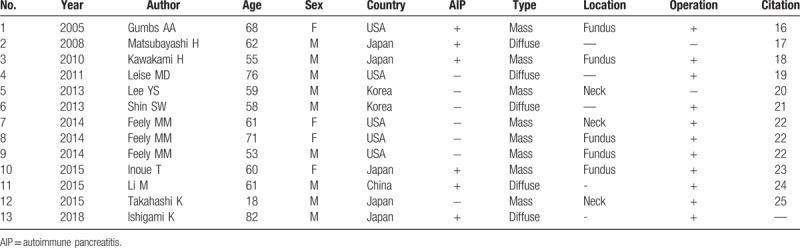
Previously reported cases of IgG4-related cholecystitis.

In the case of suspected IgG4 related cholecystitis, gallbladder cancer and other benign disease such as xanthogranulomatous cholecystitis and chronic cholecystitis, gallbladder adenomyomatosis should be considered in the differential diagnosis. Imaging findings obtained from this case suggest that presence of diffuse wall thickening with intact mucosal layer would be a helpful morphological finding to distinguish diffuse type IgG4 related cholecystitis from gallbladder cancer.

Our case has the limitation. Histological examination of surgical specimens revealed the abundant infiltration of IgG4 positive plasma cells in the gallbladder; however, it remains unclear whether IgG4-related lesion in the gallbladder existed simultaneously at the time when the patient was diagnosed with IgG4-RD. Further investigation may elucidate whether ectopic relapse occur in deceptively normal organs with potential infiltration of IgG4 positive plasma cells or actually naïve organs.

In summary, IgG4-related sclerosing cholecystitis shows various patterns in morphological examinations and sometimes mimics gallbladder cancer. A large number of patients with ectopic relapse of IgG4-RD will be required for better understanding of this manifestation of IgG4-RD.

## Acknowledgment

The authors would like to thank Genius Plus (genius.jp.net) for English language review.

## Author contributions

**Conceptualization:** Keisuke Ishigami, Masahiro Shitani, Hiroshi Nakase.

**Data curation:** Keisuke Ishigami.

**Investigation:** Yoshiharu Masaki, Ayako Ito, Noriyuki Akutsu, Motohisa Yamamoto, Masayo Motoya, Shigeru Sasaki, Hiroki Takahashi.

**Resources:** Yasutoshi Kimura, Tadashi Hasegawa, Ichiro Takemasa.

**Supervision:** Hiroshi Nakase.

**Writing – original draft:** Keisuke Ishigami, Masahiro Shitani.

**Writing – review & editing:** Hiroshi Nakase.
